# Hepatocyte-specific *Wtap* deficiency promotes hepatocellular carcinoma by activating GRB2–ERK depending on downregulation of proteasome-related genes

**DOI:** 10.1016/j.jbc.2023.105301

**Published:** 2023-09-28

**Authors:** Xinzhi Li, Chunhong Liu, Zhimin Zhang, Xueying Li, Zhicheng Yao, Yanbin Dong, Xin Wang, Zheng Chen

**Affiliations:** 1 HIT Center for Life Sciences, School of Life Science and Technology, Harbin Institute of Technology, Harbin, China; 2 Department of General surgery, The Third Affiliated Hospital of Sun Yat-sen University, Guangzhou, China; 3 Institute of Biophysics, Chinese Academy of Sciences, Beijing, China

**Keywords:** WTAP, HCC, GRB2, ERK, proteasome, inflammation

## Abstract

Wilm’s tumor 1–associating protein (WTAP), a regulatory protein of the m^6^A methyltransferase complex, has been found to play a role in regulating various physiological and pathological processes. However, the *in vivo* role of WTAP in the pathogenesis of hepatocellular carcinoma (HCC) is unknown. In this study, we have elucidated the crucial role of WTAP in HCC progression and shown that hepatic deletion of *Wtap* promotes HCC pathogenesis through activation of multiple signaling pathways. A single dose of diethylnitrosamine injection causes more and larger HCCs in hepatocyte-specific *Wtap* knockout (*Wtap*-HKO) mice than *Wtap*^flox/flox^ mice fed with either normal chow diet or a high-fat diet. Elevated CD36, IGFBP1 (insulin-like growth factor–binding protein 1), and chemokine (C–C motif) ligand 2 (CCL2) expression leads to steatosis and inflammation in the *Wtap*-HKO livers. The hepatocyte proliferation is dramatically increased in *Wtap*-HKO mice, which is due to higher activation of extracellular signal–regulated kinase (ERK) and signal transducer and activator of transcription-3 signaling pathways. Hepatic deletion of *Wtap* activates the ERK signaling pathway by increasing the protein stability of GRB2 and ERK1/2, which is due to the decreased expression of proteasome-related genes. Restoring PSMB4 or PSMB6 (two key components of the proteasome) leads to the downregulation of GRB2 and ERK1/2 in *Wtap*-HKO hepatocytes. Mechanistically, WTAP interacts with RNA polymerase II and H3K9ac to maintain expression of proteasome-related genes. These results demonstrate that hepatic deletion of *Wtap* promotes HCC progression through activating GRB2–ERK1/2-mediated signaling pathway depending on the downregulation of proteasome-related genes especially *Psmb4* and *Psmb6*.

Hepatocellular carcinoma (HCC) is a common hepatobiliary cancer with high rates of morbidity and mortality. The development and progression of HCC depend on the intricate interplay of genetic, environmental, and lifestyle factors. Most cases of HCC occur because of chronic liver damage caused by exposure to carcinogens, viral hepatitis infections, alcohol consumption, and nonalcoholic steatohepatitis (NASH) (1). The development of HCC involves various factors, such as viruses, growth factors, metabolic molecules, and inflammatory cytokines (2). These factors can activate signaling pathways like Ras–Raf–MEK (mitogen-activated protein kinase kinase)–extracellular signal–regulated kinase (ERK), MEKK1/c-Jun N-terminal kinase (JNK), PI3K/Akt/mTOR, Wnt/b-catenin, Janus kinase–signal transducer and activator of transcription-3 (STAT3), insulin-like growth factor (IGF) receptor, hepatocyte growth factor receptor (HGFR), and epidermal growth factor receptor (EGFR) that can result in the initiation, invasion, and metastasis of HCC (3, 4, 5). However, how these signaling pathways are integratively regulated is not well known. Understanding the integrative molecular mechanisms involved in the progression of HCC in response to environmental stimuli can aid in identifying early diagnostic markers or new targets for drug development.

Many disease processes such as NASH, obesity, and diabetes have been found to be regulated by m^6^A RNA writer proteins such as methyltransferase like 3 (METTL3) and Wilm’s tumor 1–associating protein (WTAP) by altering m^6^A RNA modification or chromatin accessibility (6, 7, 8, 9, 10, 11). METTL3 and WTAP have been found to be abnormally elevated in HCC (12, 13), and knocking down METTL3 or WTAP in HCC cell lines limited cell proliferation (13, 14). As a result, researchers concluded that METTL3 and WTAP increased HCC development, and that inhibiting METTL3 or WTAP may be a viable approach for HCC treatment (13, 14). However, HCC cell lines and nude animal models may not correctly reflect the activity of METTL3 and WTAP in HCC formation *in vivo*. Recently, it was demonstrated that hepatocyte-specific deletion of *Mettl3* promotes HCC in mice by activating numerous signaling pathways (15). However, the role of WTAP in HCC development *in vivo* is mainly unknown. It is also unknown if WTAP affects signaling pathways related to HCC in an integrative manner.

In this study, we used a mouse model with hepatocyte-specific *Wtap* knockout (*Wtap*-HKO) to investigate the role of WTAP in HCC progression. We have demonstrated that hepatic deletion of *Wtap* promotes the pathogenesis of HCC by activating multiple signaling pathways, at least partially through stabilizing GRB2 and ERK1/2 protein levels.

## Results

### Hepatocyte-specific deletion of *Wtap* dramatically induces HCC in mice

**Figure 1:**
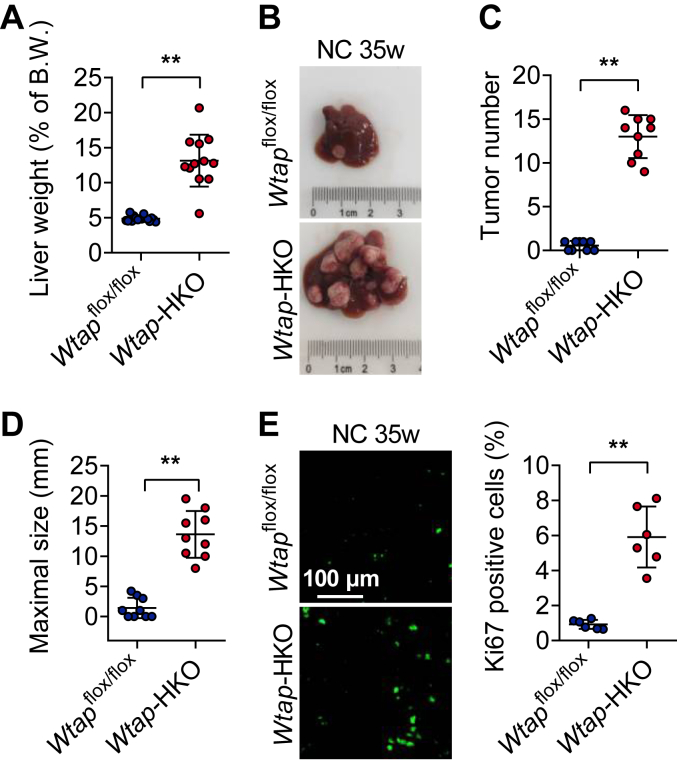
**Hepatocyte-specific *Wtap* depletion causes hepatocellular carcinoma (HCC) in mice.** At 2 weeks old, male *Wtap*-HKO mice and *Wtap*^flox/flox^ control mice were given a single intraperitoneal dose of DEN (50 mg/kg). The mice were fed an NC diet. Mice were sacrificed at the age of 35 weeks for HCC examination. *A*, the weights of the livers were measured (n = 12–16 for each group; *p* < 0.0001). *B*, representative images of mouse livers from the indicated genotype were shown. *C* and *D*, tumor number and maximal tumor size were determined (n = 9 for each group; *p* < 0.0001). *E*, representative Ki67 staining images were shown. The number of Ki67-positive cells was counted (n = 6 for each group; *p* < 0.0001). Data represent the mean ± SD. Significance was determined by unpaired two-tailed Student's *t* test analysis. ∗∗*p* < 0.01. DEN, diethylnitrosamine; NC, normal chow diet; *Wtap*-HKO, hepatocyte-specific *Wtap* knockout.

To test if WTAP influences HCC pathogenesis *in vivo*, we delivered a single intraperitoneal injection of diethylnitrosamine (DEN) (50 mg/kg) to male *Wtap*-HKO and *Wtap*^flox/flox^ mice at 2 weeks old and evaluated liver tumors at 35 weeks. *Wtap*-HKO mice had a bigger liver weight and more and larger HCCs than *Wtap*^flox/flox^ mice (Fig. 1, *A*–*D*). Cell proliferation in *Wtap*-HKO livers was considerably boosted, as evidenced by an increase in Ki67-positive cells (Fig. 1*E*). These findings show that deleting *Wtap* in the liver enhances HCC development.

### *Wtap* deletion in hepatocytes promotes HCC progression in young mice

**Figure 2:**
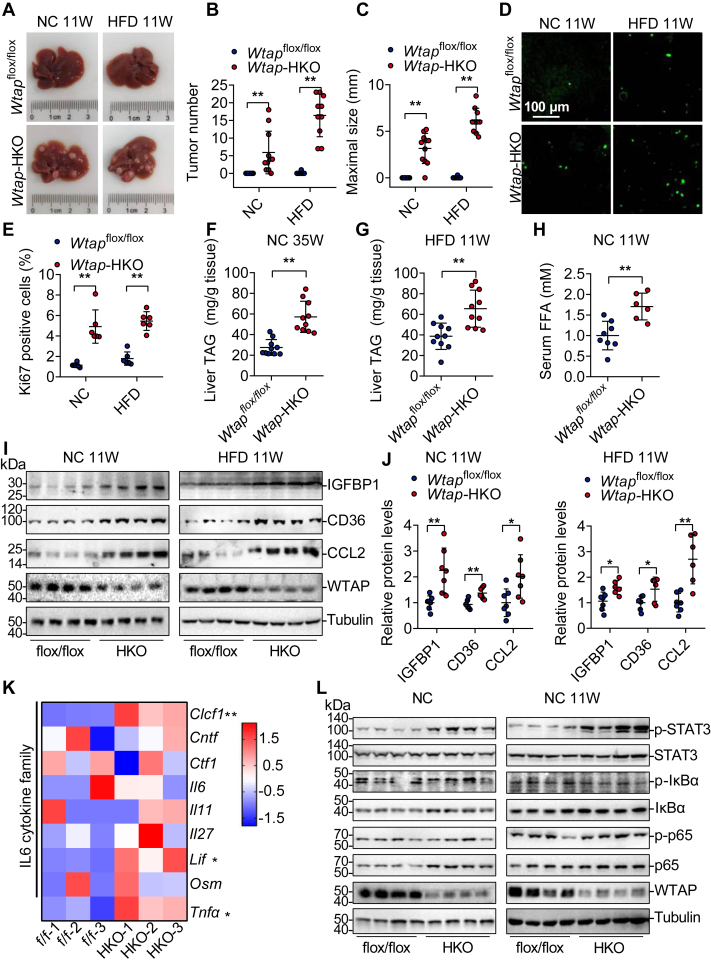
***Wtap* deletion in hepatocytes promotes hepatocellular carcinoma (HCC) progression in young mice.** At 2 weeks old, male *Wtap*-HKO mice and *Wtap*^flox/flox^ mice received a single intraperitoneal dose of DEN (50 mg/kg). Mice were fed either a normal choe (NC) diet or a high-fat diet (HFD) for 11 weeks. *A*, representative pictures of mouse livers from the indicated genotype are shown. *B*, tumor number was counted (n = 10–12 per group; NC: *p* = 0.0026; HFD: *p* < 0.0001). *C*, maximal tumor size was measured (n = 10–12 per group; NC: *p* < 0.0001; HFD: *p* < 0.0001). *D*, representative Ki67 staining photographs were shown. *E*, the number of Ki67-positive cells was counted (n = 6 per group; NC: *p* = 0.0002; HFD: *p* < 0.0001). *F* and *G*, triglyceride (TAG) levels in the livers of *Wtap*-HKO mice and *Wtap*^flox/flox^ mice fed an NC diet for 35 weeks or an HFD for 11 weeks were assessed (n = 10 per group; *F*: *p* < 0.0001; *G*: *p* = 0.0012). *H*, serum-free fatty acid levels in *Wtap*-HKO mice and *Wtap*^flox/flox^ mice fed with an NC diet for 11 weeks (n = 6–8 per group; *p* = 0.0022). *I* and *J*, immunoblotting of IGFBP1, CD36, CCL2, WTAP, and Tubulin in the livers of *Wtap*-HKO and *Wtap*^flox/flox^ mice fed NC or HFD for 11 weeks following DEN treatment (n = 7 per group; n = 4 for representative images; NC: IGFBP1, *p* = 0.0033; CD36, *p* = 0.0013; and CCL2, *p* = 0.019; HFD: IGFBP1, *p* = 0.0115; CD36, *p* = 0.0264; and CCL2, *p* = 0.0011). *K*, heatmap of interleukin 6 (IL6) cytokine family (*Clcf1*, *Cntf*, *Ctf1*, *Il6*, *IL11*, *Il27*, *Lif*, and *Osm*) and *Tnfa* in *Wtap*-HKO and *Wtap*^flox/flox^ livers (n = 3 for each group, RNA-Seq data GSE168850). *L*, immunoblotting analysis of p-STAT3, STAT3, p-p65, p65, p-IκBα, IκBα, WTAP, and Tubulin in the livers of NC-fed *Wtap*-HKO and *Wtap*^flox/flox^ mice pretreated with or without DEN (n = 7 for each group; n = 3 for representative images). ∗*p*< 0.05; ∗∗*p*< 0.01. Data represent the mean ± SD. CCL2, chemokine (C–C motif) ligand 2; DEN, diethylnitrosamine; IGFBP1, insulin-like growth factor–binding protein 1; *Wtap*-HKO, hepatocyte-specific *Wtap* knockout.

A recent study found that WTAP controls nonalcoholic fatty liver disease and NASH progression, and *Wtap*-HKO mice showed NASH-like phenotypes in both normal chow (NC) and NASH diet–fed circumstances (7). We wanted to see if *Wtap*-HKO mice develop HCC under short-term feeding circumstances. After a short period of feeding (11 weeks) of either NC or high-fat diet (HFD) following DEN injection, *Wtap*-HKO mice developed considerably more and larger HCC tumors, whereas *Wtap*^flox/flox^ mice did not (Fig. 2, *A*–*C*). Cell proliferation was consistently increased in both NC- and HFD-fed DEN-treated *Wtap*-HKO mice, as seen by increased Ki67-positive cells (Fig. 2, *D* and *E*). These findings show that hepatic *Wtap* deletion exacerbates DEN-induced HCC development under short-term feeding circumstances.

We then investigated whether hepatic *Wtap* deletion accelerates DEN-induced HCC progression by enhancing liver steatosis and inflammation. Following DEN treatment, *Wtap*-HKO mice showed higher liver triglyceride levels than *Wtap*^flox/flox^ mice under both NC diet (35 weeks) and HFD (11 weeks) feeding circumstances (Fig. 2, *F* and *G*), which is likely related to elevated serum-free fatty acid (Fig. 2*H*) and expression of CD36 and IGFBP1 (insulin-like growth factor–binding protein 1) (Fig. 2, I and *J*) (7). A tumor-promoting inflammatory microenvironment is also critical in the development of HCC. The expression of important cytochemokines such as chemokine (C–C motif) ligand 2 (CCL2) and tumor necrosis factor alpha (TNFα) was shown to be dramatically enhanced in *Wtap*-HKO mice (Fig. 2, I–*K*) (7). Some members of the interleukin (IL) 6 cytokine family, such as CLCF1 and LIF, were elevated, but others, such as CNTF, CTF1, IL6, IL11, IL27, and OSM, were not (Fig. 2*K*). Elevated CLCF1 and LIF levels may help activate STAT3 (16, 17). STAT3 phosphorylation was dramatically increased in *Wtap*-HKO mice (Figs. 2*L* and S1), contributing to higher cell proliferation. The classical TNF signaling cascade (phosphorylation of IκBα and p65) was not elevated in *Wtap*-HKO mice treated with or without DEN (Figs. 2*L* and S1).

### *Wtap* deletion in hepatocytes activates the GRB2–ERK signaling pathway

**Figure 3:**
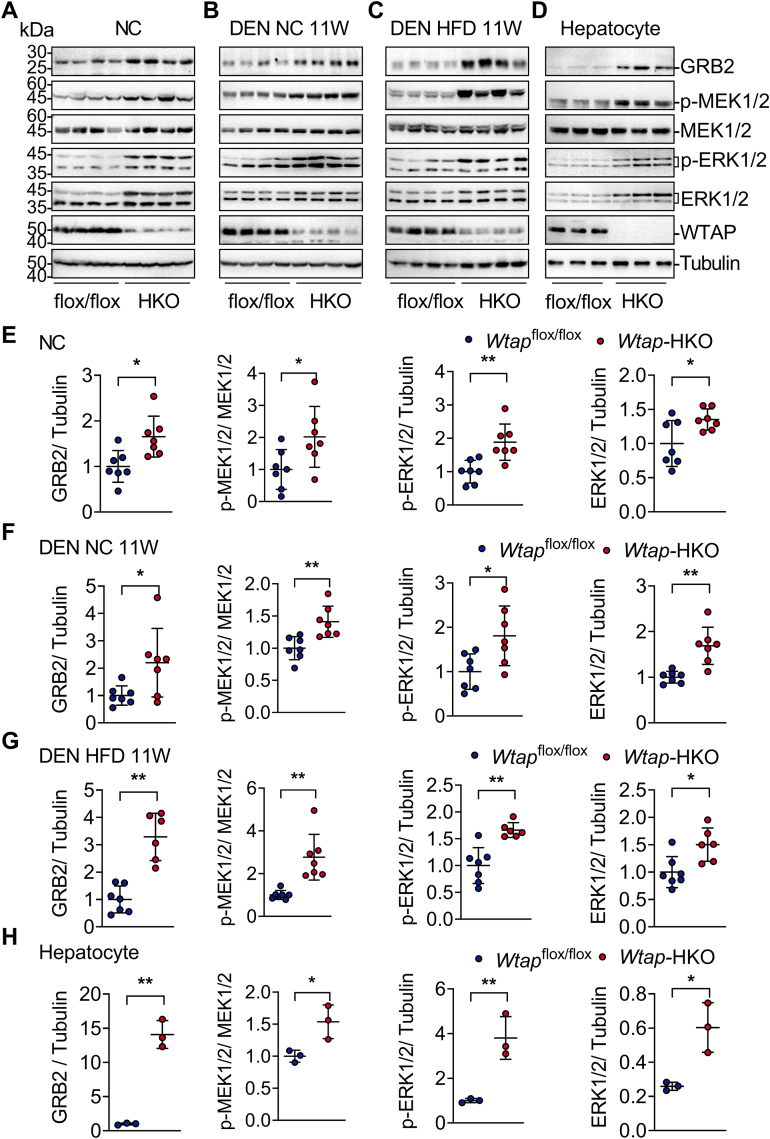
***Wtap* deletion in hepatocytes promotes the GRB2–MEK1/2–ERK1/2 signaling pathway.***A* and *E*, immunoblotting analysis of GRB2, p-MEK1/2, MEK1/2, p-ERK1/2, ERK1/2, WTAP, and Tubulin in the livers of *Wtap*-HKO and *Wtap*^flox/flox^ mice fed NC for 8 weeks (n = 7 for each group; n = 4 for representative images; GRB2/Tubulin: *p* = 0.0101; p-MEK1/2/MEK1/2: *p* = 0.0351; p-ERK1/2/Tubulin: *p* = 0.0034; ERK1/2/Tubulin: *p* = 0.0272). *B*, *C*, *F*, and *G*, immunoblotting analysis of GRB2, p-MEK1/2, MEK1/2, p-ERK1/2, ERK1/2, WTAP, and Tubulin in the livers of *Wtap*-HKO and *Wtap*^flox/flox^ mice fed with NC or HFD for 11 weeks following DEN treatment (n = 7 for each group; n = 4 for representative images; DEN NC 11W: GRB2/Tubulin: *p* = 0.0314; p-MEK1/2/MEK1/2: *p* = 0.0036; p-ERK1/2/Tubulin: *p* = 0.0183; ERK1/2/Tubulin: *p* = 0.0011; DEN HFD 11W: GRB2/Tubulin: *p* < 0.0001; p-MEK1/2/MEK1/2: *p* = 0.0011; p-ERK1/2/Tubulin: *p* = 0.0009; and ERK1/2/Tubulin: *p* = 0.011). *D* and *H*, GRB2, p-MEK1/2, MEK1/2, p-ERK1/2, ERK1/2, WTAP, and Tubulin immunoblotting were performed in primary hepatocytes isolated from *Wtap*-HKO and *Wtap*^flox/flox^ mice (n = 3; GRB2/Tubulin: *p* = 0.0004; p-MEK1/2/MEK1/2: *p* = 0.0289; p-ERK1/2/Tubulin: *p* = 0.0072; and ERK1/2/Tubulin: *p* = 0.0154). The immunoblotting assays were done three times independently with similar results. Differences between two groups were analyzed by Student's *t* tests. ∗*p* < 0.05; ∗∗*p* < 0.01. Data represent the mean ± SD. DEN, diethylnitrosamine; ERK1/2, mitogen-activated protein kinase kinase 1/2; HFD, high-fat diet; MEK1/2, mitogen-activated protein kinase kinase 1/2; NC, normal chow diet; WTAP, Wilm’s tumor 1–associating protein; *Wtap*-HKO, hepatocyte-specific *Wtap* knockout.

In addition to the elevated STAT3 signaling pathway, *Wtap*-HKO mice have considerably increased ERK phosphorylation (Fig. 3, *A*, *B*, *C*, *E*, *F*, and *G*), which contributes to higher cell proliferation. Increased GRB2 and p-MEK1/2 levels contributed to ERK activation (Fig. 3, *A*, *B*, *C*, *E*, *F*, and *G*). *Wtap*-HKO livers had higher amounts of ERK1/2 protein (Fig. 3, *A*, *B*, *C*, *E*, *F*, and *G*), which may contribute to ERK activation. However, upstream receptor activation (EGFR and HGFR/Met) was intact or decreased (Fig. S2, *A*–*F*), showing that EGFR and HGFR/Met are unlikely to contribute to GRB2–MEK–ERK signaling pathway activation in *Wtap*-HKO livers. Gab1 and p-B-Raf levels were modestly elevated in *Wtap*-HKO livers, which may also contribute to ERK signaling pathway activation (Fig. S2, *A*–*E*). GRB2, p-MEK1/2, p-ERK1/2, and ERK1/2 levels were likewise increased in isolated *Wtap*-HKO hepatocytes (Fig. 3, *D* and *H*). These findings show that hepatic *Wtap* deletion exacerbates DEN-induced HCC formation, at least in part, *via* activating the GRB2–ERK signaling pathway.

### Hepatocyte-specific deficiency of *Wtap* increases protein stability of GRB2 and ERK1/2

**Figure 4:**
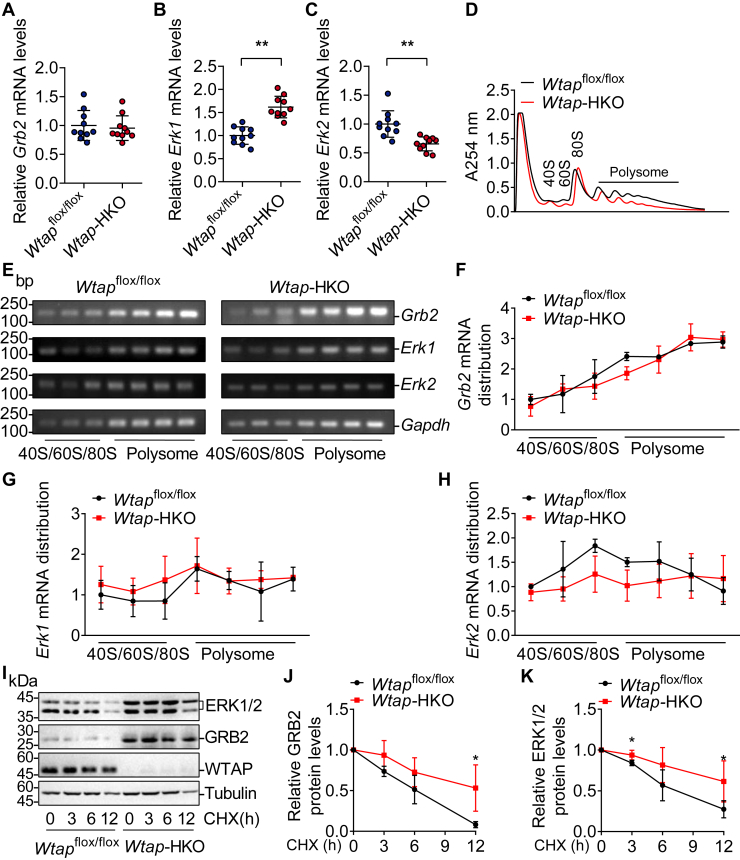
***Wtap* deficiency in hepatocytes increases the protein stability of GRB2 and ERK1/2.***A*–*C*, RT–quantitative PCR (qPCR) was used to determine the mRNA levels of *Grb2* and *Erk1/2* in the livers of *Wtap*-HKO and *Wtap*^flox/flox^ mice (n = 10 mice for each group; *Grb2*: *p* = 0.6707; *Erk1*: *p* < 0.0001; *Erk2*: *p* = 0.0005). *D*–*H*, sucrose gradient centrifugation was used to identify the fractions 40s, 60s, 80s, and polysome in the livers of *Wtap*-HKO and *Wtap*^flox/flox^ mice. Semiquantitative RT–PCR was used to determine the relative distribution of *Grb2* and *Erk1/2* mRNAs in these fractions (n = 3 mice for each group). *I*–*K*, primary hepatocytes were extracted from *Wtap*-HKO and *Wtap*^flox/flox^ mice and subsequently exposed to cycloheximide (CHX; 3 μg/ml) for varying periods (0, 3, 6, and 12 h). Immunoblotting was used to determine the amounts of ERK1/2, GRB2, WTAP, and Tubulin protein. ERK1/2 and GRB2 were then quantified using ImageJ and normalized to their protein levels at 0 point, respectively (n = 4 mice for each group; GRB2: 3 h, *p* = 0.0822; 6 h, *p* = 0.1367; 12 h, *p* = 0.0203; ERK1/2: 3 h, *p* = 0.0281; 6 h, *p* = 0.1328; 12 h, *p* = 0.0474). Differences between two groups were analyzed by Student's *t* tests. ∗*p* < 0.05 and ∗∗*p* < 0.01. Data represent the mean ± SD. ERK1/2, extracellular signal–regulated kinase 1/2; qPCR, quantitative PCR; WTAP, Wilm’s tumor 1–associating protein; *Wtap*-HKO, hepatocyte-specific *Wtap* knockout.

We investigated how *Wtap* deficiency in hepatocytes raises GRB2 and ERK1/2 protein levels. GRB2 and ERK1/2 protein levels were observed to be elevated in the livers and isolated hepatocytes of *Wtap*-HKO mice (Fig. 3, *A-H*). Only *Erk1* mRNA levels were modestly elevated, whereas *Grb2* and *Erk2* mRNA levels were not (Fig. 4, *A*–*C*), implying that *Wtap* deficiency promotes translational efficiency or protein stability. We measured translational efficiency in *Wtap*^flox/flox^ and *Wtap-*HKO mice by sucrose gradient centrifugation of *Wtap*^flox/flox^ and *Wtap-*HKO polysome fractions, and RT–PCR of endogenous mRNA levels of *Grb2*, *Erk1*, and *Erk2* in different ribosome fractions. As shown in Figure 4, *D*–*H*, the relative distribution of *Grb2*, *Erk1*, and *Erk2* mRNAs in polysome fractions was not altered in *Wtap*-HKO livers, showing that hepatic deletion of *Wtap* had no effect on the translational efficiency of *Grb2*, *Erk1*, and *Erk2* mRNA. GRB2 and ERK1/2 protein stability was also assessed. GRB2 and ERK1/2 protein stability was considerably elevated in isolated hepatocytes from *Wtap-*HKO mice (Fig. 4, I–*K*). These findings show that hepatic deficiency of *Wtap* increases the protein stability of GRB2 and ERK1/2.

### Hepatocyte-specific deficiency of *Wtap* reduces the expression of proteasome-related genes, which contributes to higher protein stability of GRB2 and ERK

**Figure 5:**
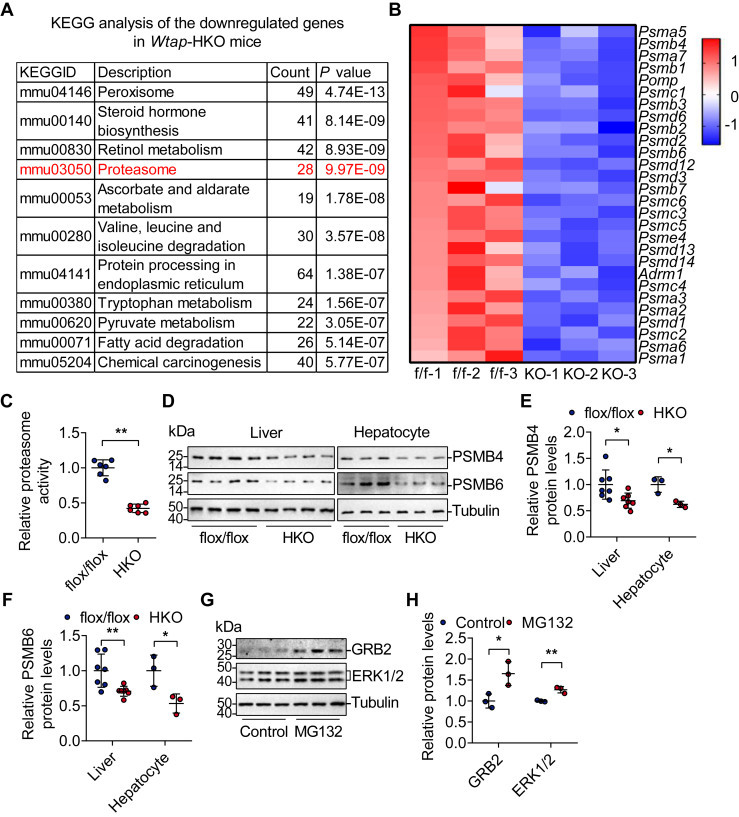
***Wtap* deficiency in hepatocytes reduces the expression of proteasome-associated genes, which contributes to higher protein stability of GRB2 and ERK.***A* and *B*, at 8 weeks old, RNA-Seq analysis was done in the livers of *Wtap*^flox/flox^ and *Wtap-*HKO mice (n = 3 for each group, GSE168850). Kyoto Encyclopedia of Genes and Genomes pathway analysis of the downregulated genes in *Wtap-*HKO mice (*A*). A heatmap of downregulated proteasome-related genes was displayed (*B*). *C*, proteasome activity assay was measured in the livers of *Wtap*^flox/flox^ and *Wtap-*HKO mice (n = 6 mice per group; *p* < 0.0001). *D*–*F*, PSMB4 and PSMB6 protein levels in *Wtap*^flox/flox^ and *Wtap-*HKO livers (n = 7 mice per group; PSMB4: *p* = 0.0233; PSMB6: *p* = 0.0085) and hepatocytes (n = 3 independent cell samples for each group; PSMB4: *p* = 0.0151; PSMB6: *p* = 0.0363) were determined by immunoblotting. *G* and *H*, primary hepatocytes were isolated from WT mice and treated with or without MG132. GRB2, ERK1/2, and Tubulin protein levels were determined by immunoblotting and quantified using ImageJ (n = 3 independent samples for each group; GRB2: *p* = 0.0262; ERK1/2: *p* = 0.0038). Differences between two groups were analyzed by Student's *t* tests. ∗*p*< 0.05 and ∗∗*p*< 0.01. Data represent the mean ± SD. ERK, extracellular signal–regulated kinase.

To determine how hepatic deficiency of *Wtap* increases protein stability of GRB2 and ERK1/2, we analyzed our previous RNA-Seq data (GSE168850) (7) and noticed that downregulated genes were associated with the proteasome (Fig. 5*A*), and 28 proteasome-associated genes were downregulated (Fig. 5*B*), which was confirmed by RT–quantitative PCR (qPCR) (Fig. S3). Proteasome activity was significantly decreased in *Wtap-*HKO livers (Fig. 5*C*). This was also observed in *Mettl3-*HKO livers (18). Among the downregulated proteasome components, PSMB4 and PSMB6 were involved in METTL3-mediated MKK4–JNK protein stability (18). PSMB4 and PSMB6 protein levels were also considerably reduced in *Wtap-*HKO livers or primary hepatocytes (Fig. 5, *D*–*F*). Furthermore, MG132, an inhibitor of the proteasome, raised GRB2 and ERK1/2 protein levels in primary hepatocytes (Fig. 5, *G* and *H*), demonstrating that decreased proteasome activity leads to higher GRB2 and ERK1/2 protein levels.

### Restoring proteasome activity *via* overexpression of PSMB4 or PSMB6 decreases GRB2 and ERK1/2 protein levels in *Wtap-*HKO hepatocytes

**Figure 6:**
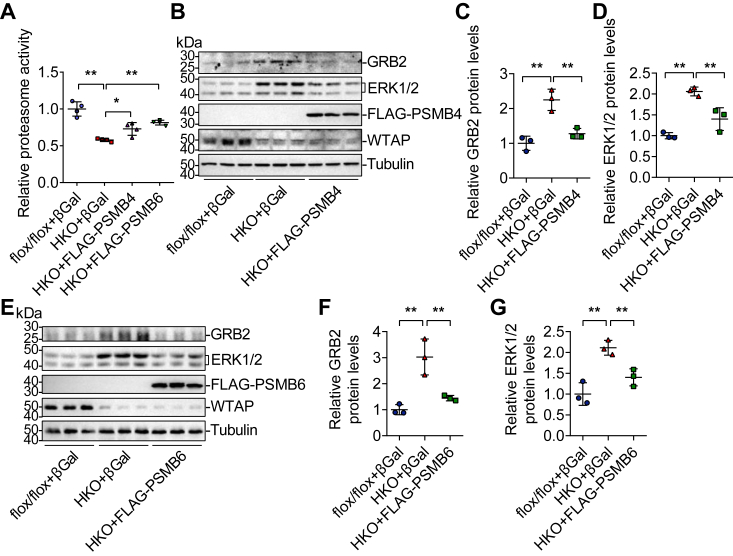
**Restoring proteasome activity *via* overexpression of PSMB4 or PSMB6 decreases GRB2 and ERK1/2 protein levels in *Wtap-*HKO hepatocytes.** Primary hepatocytes were isolated from *Wtap*-HKO and *Wtap*^flox/flox^ mice and then infected with Ad-βGal, Ad-FLAG-PSMB4, and Ad-FLAG-PSMB6 adenoviruses, respectively. *A*, proteasome activity was measured (n = 4 independent samples for each group; HKO + βGal *versus* flox/flox + βGal: *p* < 0.0001; HKO + βGal *versus* HKO + FLAG-PSMB4: *p* = 0.0204; HKO + βGal *versus* HKO + FLAG-PSMB6: *p* = 0.0003). *B*–*D*, immunoblotting and ImageJ analysis were used to determine the protein levels of GRB2, ERK1/2, FLAG, WTAP, and Tubulin in βGal- and Ad-FLAG-PSMB4-overexpressing hepatocytes, respectively. GRB2 and ERK1/2 levels were normalized to Tubulin levels (n = 3 independent samples for each group; GRB2: HKO + βGal *versus* flox/flox + βGal, *p* = 0.0006; HKO + βGal *versus* HKO + FLAG-PSMB4, *p* = 0.002; ERK1/2: HKO + βGal *versus* flox/flox + βGal, *p* = 0.0003; HKO + βGal *versus* HKO + FLAG-PSMB4, *p* = 0.0034). *E*–*G*, immunoblotting and ImageJ analysis were used to determine the protein levels of GRB2, ERK1/2, FLAG, WTAP, and Tubulin in βGal- and Ad-FLAG-PSMB4-overexpressing hepatocytes, respectively. GRB2 and ERK1/2 levels were normalized to Tubulin levels (n = 3 independent samples for each group; GRB2: HKO + βGal *versus* flox/flox + βGal, *p* = 0.001; HKO + β Gal *versus* HKO + FLAG-PSMB6, *p* = 0.0035; ERK1/2: HKO + βGal *versus* flox/flox + βGal, *p* = 0.0009; HKO + βGal *versus* HKO + FLAG-PSMB6, *p* = 0.0078). These experiments were conducted three times independently with similar results. Differences between three groups were analyzed by one-factor ANOVA, and the least significance difference (LSD) *t* test. ∗*p* < 0.05; ∗∗*p* < 0.01. Data represent the mean ± SD. ERK1/2, extracellular signal–regulated kinase 1/2; WTAP, Wilm’s tumor 1–associating protein; *Wtap*-HKO, hepatocyte-specific *Wtap* knockout.

We investigated whether restoring proteasome activity *via* PSMB4 or PSMB6 overexpression could correct the increased protein levels of GRB2 and ERK1/2 in *Wtap-*HKO hepatocytes. We isolated primary hepatocytes from *Wtap*^flox/flox^ and *Wtap-*HKO mice, respectively. Hepatocytes were infected with an equal amount of Ad-βGal, Ad-FLAG-PSMB4, or Ad-FLAG-PSMB6 adenoviruses overnight. Proteasome activity was significantly decreased (Fig. 6*A*), whereas the GRB2 and ERK1/2 protein levels were upregulated in *Wtap-*HKO hepatocytes (Fig. 6, *B*–*G*). PSMB4 or PSMB6 overexpression restored proteasome activity (Fig. 6*A*), which reversed the upregulation of GRB2 and ERK1/2 in *Wtap-*HKO hepatocytes (Fig. 6, *B*–*G*). These findings suggest that hepatic deficiency of *Wtap* reduces the expression of proteasome-related genes especially *Psmb4* and *Psmb6*, which contributes to the increased protein stability of GRB2 and ERK1/2.

### WTAP binds to the promoters of proteasome-related genes and promotes their expression

**Figure 7:**
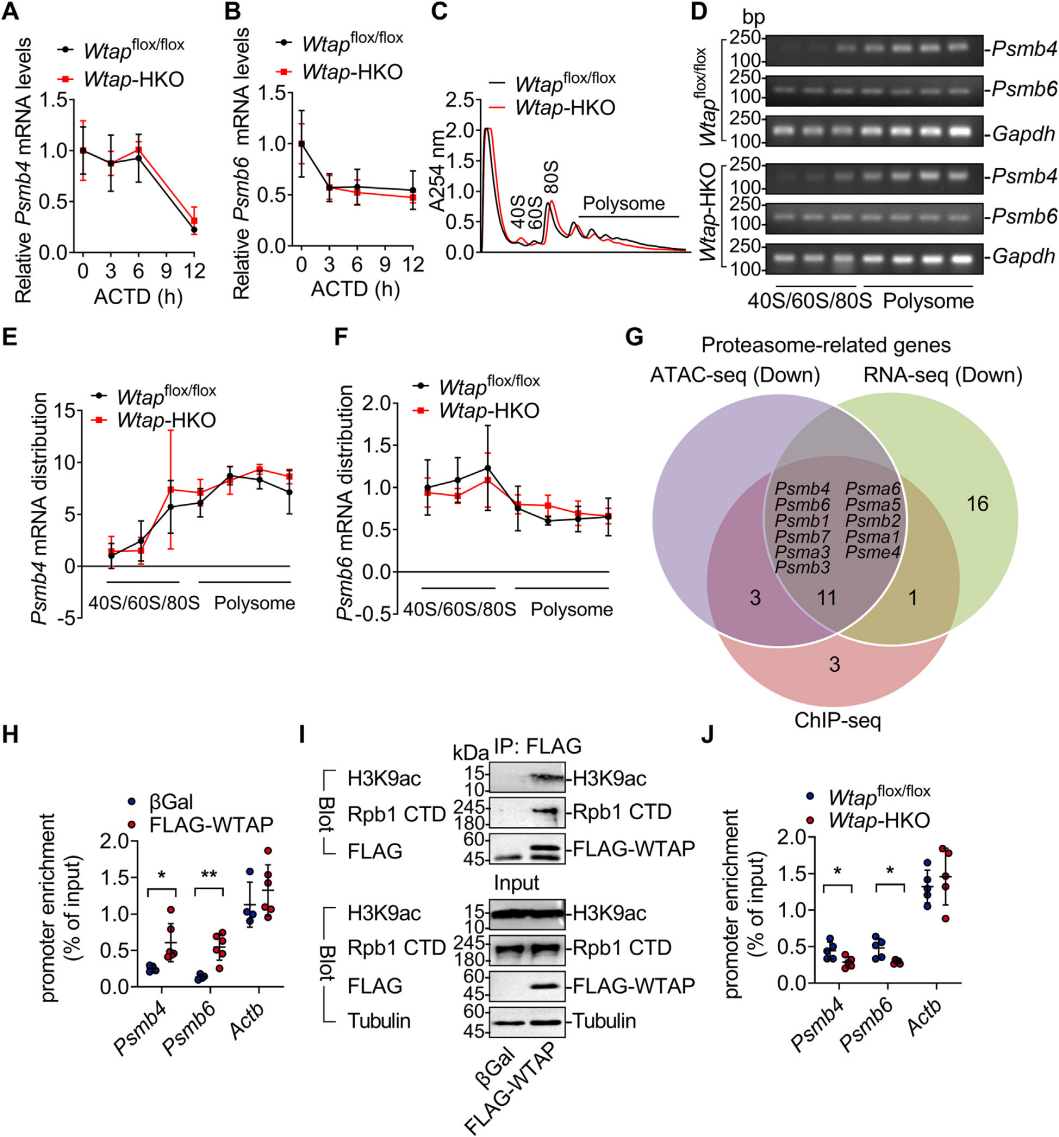
**WTAP binds to the promoters of proteasome-related genes and promotes their expression**. *A* and *B*, primary hepatocytes were isolated from *Wtap*-HKO and *Wtap*^flox/flox^ mice and then treated with ACTD (10 μg/ml) for different times (0, 3, 6, and 12 h). RT–qPCR was used to determine *Psmb4* and *Psmb6* mRNA levels (n = 6 independent cell samples for each group). *C*–*F*, sucrose gradient centrifugation was used to identify the fractions of 40s, 60s, 80s, and polysome in the livers of *Wtap*-HKO and *Wtap*^flox/flox^ mice. Semiquantitative RT–PCR was used to determine the relative distribution of *Psmb4* and *Psmb6* mRNAs in these fractions (n = 3 mice for each group). *G*, a combined analysis of the ATAC-Seq (GSE168945), RNA-Seq (GSE168850), and ChIP-Seq (GSE198023) data revealed that WTAP binds to the promoters of 11 proteasome-related genes, including *Psmb4*, *Psmb6*, *Psmb1*, *Psmb7*, *Psma3*, *Psmb3*, *Psma6*, *Psma5*, *Psmb2*, *Psma1*, and *Psme4*, and their expression was downregulated in *Wtap-*HKO livers. *H*, ChIP–qPCR was used to assess FLAG-WTAP binding to the promoters of *Psmb4*, *Psmb6*, and *Actb* genes in the primary hepatocytes infected with Ad-FLAG-WTAP or βGal adenovirus. FLAG beads were used to immunoprecipitate the hepatocyte samples. Immunoprecipitated DNA was extracted for qPCR analysis (n = 4–6 for each group; *Psmb4*: *p* = 0.0302; *Psmb6*: *p* = 0.0022; *Actb*: *p* = 0.3862). *I*, primary hepatocytes were isolated from *WT* mice and then infected with βGal and FLAG-WTAP adenoviruses, respectively. Total cell lysates were treated with DNase1 (200 U/ml) at 37 °C for 30 min. FLAG beads were used to immunoprecipitate the cell lysates, which were subsequently immunoblotted with anti-H3K9ac anti-Rpb1 CTD and anti-FLAG antibodies. This experiment was conducted twice independently with similar results. *J*, ChIP–qPCR was used to assess RNA polymerase II (Rpb1 CTD) levels binding to the promoters of *Psmb4*, *Psmb6*, and *Actb* genes in the livers of *Wtap*^flox/flox^ and *Wtap-*HKO mice at 8 weeks old (n = 5 mice for each group; *Psmb4*: *p* = 0.0344; *Psmb6*: *p* = 0.0131; *Actb*: *p* = 0.5189). Differences between two groups were analyzed by Student's *t* tests. ∗*p* < 0.05 and ∗∗*p* < 0.01. Data represent the mean ± SD. ChIP, chromatin immune oprecipitation; CTD, C-terminal domain; qPCR, quantitative PCR; WTAP, Wilm’s tumor 1–associating protein; *Wtap*-HKO, hepatocyte-specific *Wtap* knockout.

WTAP binds to METTL3 and acts as a regulator of the m^6^A RNA methyltransferase complex (12). We examined prior methylated RNA immunoprecipitation sequencing data (GSE192884) from the livers of *Wtap*^flox/flox^ and *Wtap-*HKO mice (7) and discovered that the m^6^A peaks of certain transcripts encoding proteasome components, including *Psmb4* and *Psmb6*, were reduced. Then we looked to see if *Psmb4* and *Psmb6* mRNA stability and translational efficiency were affected in *Wtap-*HKO mice. *Psmb4* and *Psmb6* mRNA stability and translational efficiency were not altered in *Wtap-*HKO mice (Fig. 7, *A*–*F*). WTAP has also been linked to gene transcription regulation (7). We examined earlier ATAC-Seq data (GSE168945) from the livers of *Wtap*^flox/flox^ and *Wtap-*HKO mice and discovered 14 proteasome-related genes with downregulated peaks (Fig. 7*G*). According to a combined analysis of the ATAC-Seq and RNA-Seq data, 11 proteasome-related genes, including *Psmb4*, *Psmb6*, *Psmb1*, *Psmb7*, *Psma3*, *Psmb3*, *Psma6*, *Psma5*, *Psmb2*, *Psma1*, and *Psme4*, were downregulated in *Wtap-*HKO livers (Fig. 7*G*). These findings suggest that hepatic deletion of *Wtap* lowers transcription of these genes. WTAP binds to gene promoters, according to our recent findings (7). We examined our prior chromatin immunoprecipitation (ChIP)-Seq data (GSE198023) generated from FLAG-WTAP adenovirus-infected hepatocytes and found that these 11 proteasome-related genes (*Psmb4*, *Psmb6*, *Psmb1*, *Psmb7*, *Psma3*, *Psmb3*, *Psma6*, *Psma5*, *Psmb2*, *Psma1*, and *Psme4*) were in the list of genes having peaks (Fig. 7*G*). Furthermore, WTAP binds to the promoters of *Psmb4* and *Psmb6* but does not bind to the promoter of *β-actin* (*Actb*) as detected by ChIP in the FLAG-WTAP-overexpressing hepatocytes (Fig. 7*H*). These findings imply that WTAP binds to the promoters of proteasome-related genes and promotes their expression.

METTL14–METTL3 has been demonstrated to influence gene expression *via* binding to RNA polymerase II and active histone modifications such as H3K36me3, H3K9ac, and H3K27ac (18, 19). WTAP interacted with RNA polymerase II (Rpb1 C-terminal domain [CTD]) and H3K9ac in primary hepatocytes (Fig. 7*I*). The binding of RNA polymerase II (Rpb1 CTD) to genes of *Psmb4* and *Psmb6* was reduced in the livers of *Wtap-*HKO mice, whereas the binding of RNA polymerase II (Rpb1 CTD) to *Actb* was unaffected (Fig. 7*J*). These findings suggest that WTAP is essential for maintaining the expression of proteasome components through interacting with RNA polymerase II and H3K9ac.

## Discussion

Many physiological and pathological processes have been shown to be regulated by m^6^A writer proteins (METTL3/METTL14/WTAP), including circadian rhythms (20), DNA damage response (21), stem cell differentiation (22, 23), brown adipose tissue development (8, 9), NASH (6, 7), and diabetes (10, 24), in both m^6^A-dependent and -independent manners. In this study, we found that deleting *Wtap* in hepatocytes enhances HCC development by activating numerous signaling pathways, particularly the GRB2–ERK signaling pathway.

In *Wtap*-HKO livers, many signaling pathways contribute to the development of HCC. Hepatic deletion of *Wtap* induces lipolysis in white adipose tissue and NASH in the liver (7), exacerbating DEN-induced HCC development. IGFBP1 has been demonstrated to raise serum-free fatty acid by stimulating lipolysis in white adipose tissue. *Wtap*-HKO mice have abnormally raised CD36 levels, which contributes to the enhanced steatosis in both NC diet– and HFD-fed *Wtap*-HKO mice. Inflammation is another factor that contributes to HCC development. TNFα and CCL2 levels were considerably increased in *Wtap*-HKO mice (7), which promotes HCC growth. WTAP directly regulates the transcription of *Igfbp1*, *Cd36*, and *Ccl2*, which increases the development of NASH (7). Elevated CLCF1 and LIF levels may lead to STAT3 activation, which enhances tumor cell proliferation, including HCC (25). Hepatic deletion of *Wtap* does not increase the canonical TNF signaling cascade (phosphorylation of IκBα and p65), implying that WTAP modulates cytochemokine production independent of the TNF signaling pathway.

The phosphorylation of ERK1/2 is significantly raised in both NC- and HFD-fed *Wtap*-HKO mice, in addition to IGFBP1, CD36, CCL2, and p-STAT3. High ERK signaling pathway activation promotes tumor cell growth, including HCC (25). ERK activation may be linked to upstream signaling molecules such as GRB2 and p-MEK1/2 being activated in *Wtap*-HKO livers. *Wtap*-HKO livers have considerably greater ERK1/2 protein levels, which may lead to higher p-ERK1/2 levels. The phosphorylation of EGFR and Met is not elevated in *Wtap*-HKO livers, indicating that WTAP regulates ERK signaling pathway activation independent of EGFR and Met.

Our investigation focused on how WTAP deficiency in hepatocytes affects GRB2 and ERK1/2 activation and expression. Hepatic deletion of *Wtap* does not affect translational efficiency of *Grb2* and *Erk1/2* mRNA, whereas *Wtap*-HKO livers have a lower overall global translational efficiency. Instead, protein stability of GRB2 and ERK1/2 is elevated in *Wtap*-HKO livers. RNA-Seq identifies multiple downregulated proteasome-related genes (including *Psmb4* and *Psmb6*) in *Wtap-*HKO mice. GRB2 and ERK1/2 protein levels are increased when the proteasome is inhibited by MG132. Both PSMB4 and PSMB6 overexpression reduce GRB2 and ERK1/2 protein levels in *Wtap-*HKO hepatocytes. These findings suggest that deleting WTAP in the liver raises GRB2 and ERK1/2 protein levels through downregulating proteasome-related genes, particularly *Psmb4* and *Psmb6*. *Mettl3*-HKO livers also have higher MKK4/JNK, GRB2, p-ERK, and ERK protein levels, which are likely attributable to downregulation of proteasome-related genes (15, 18), demonstrating that WTAP and METTL3 share the same molecular processes. We noticed that downregulated proteasome activity in *Mettl3*-HKO and *Wtap-*HKO livers does not increase all the proteins. The detailed molecular mechanisms of the selection need further investigation. Ubiquitin-mediated proteolysis may be also involved in this process.

WTAP binds to METTL3 and acts as a regulator of the m^6^A RNA methyltransferase complex (12). *Wtap* deletion in hepatocytes significantly alters gene expression profiles (7). Some alterations are dependent on m^6^A modification, whereas others are dependent on chromatin accessibility that is independent of m^6^A modification (7). Although *Psmb4* and *Psmb6* are two of the m^6^A peak downregulated genes, their mRNA stability and translational efficiency remain unchanged. Instead, chromatin accessibility in the promoters of *Psmb4* and *Psmb6* is reduced. The binding of RNA polymerase II to the promoters of *Psmb4* and *Psmb6* is consistently reduced in *Wtap*-HKO livers. WTAP interacts with RNA polymerase II and H3K9ac on a molecular level. METTL14 and METTL3 have also been demonstrated to influence gene expression by binding to RNA polymerase II and H3K36me3 (19). METTL3 and WTAP have also been demonstrated to bind to gene promoters and influence gene transcription *via* distinct mechanisms (6, 7, 26). These findings suggest that WTAP is essential for maintaining the expression of proteasome components (including PSMB4 and PSMB6) through binding to RNA polymerase II and active histone modifications such as H3K9ac.

Loss of m^6^A writer protein WTAP or METTL3 in hepatocytes enhances hepatic steatosis, inflammation, and activation of STAT3 and ERK signaling pathways, which exacerbates the course of DEN-induced HCC. *Wtap* or *Mettl3* deletion in other tissues or cell types, such as brown adipose tissue and islet β cells, results in tissue/cell injury and illness (6, 8, 9, 10, 11, 18). These findings suggest that the m^6^A writer proteins WTAP and METTL3 are required for maintaining tissue homeostasis. On the other hand, WTAP and METTL3 are abundantly expressed in HCC and are necessary for tumor cell proliferation (13, 14). *Wtap* or *Mettl3* knockdown in HCC cell lines reduces HCC cell proliferation, cell survival, migration, and colony formation, resulting in the inhibition of HCC tumorigenicity in nude mouse models (13, 14). These findings further suggest that hepatic WTAP and METTL3 may play separate roles in HCC development. Downregulation or inactivation of WTAP or METTL3 during the initial stage, for example, may induce HCC, whereas upregulation or activation of WTAP or METTL3 during the later stage may accelerate HCC. This idea is supported by current data. Nuclear WATP or METTL3 downregulation has been linked to NASH (6, 7), and hepatic *Wtap* or *Mettl3* deletion enhances liver damage, nonalcoholic fatty liver disease, and HCC initiation (6, 7, 27), whereas WTAP and METTL3 are abnormally elevated in HCC (13, 14), promoting HCC progression.

In conclusion, our findings show that hepatic *Wtap* deletion enhances HCC progression by activating GRB2–ERK1/2-mediated signaling pathway dependent on the downregulation of proteasome-related genes, particularly *Psmb4* and *Psmb6*.

## Experimental procedures

### Animal experiments

The Guide for the Care and Use of Laboratory Animals was strictly followed during animal research. The animal experiment protocols were approved by Institutional Animal Care and Use Committee (IACUC) of the Harbin Institute of Technology with authorization number IACUC-2018004. Mice were kept on a 12 h light/12 h dark cycle and fed various diets with free access to water. *Wtap*^flox/flox^ mice have been previously described (7, 9, 11). Briefly, exon 4 of *Wtap* gene was flanked by two loxp sites using the CRISPR–Cas9 technique. *Wtap*^flox/flox^ mice were crossed with *Alb*-Cre mice to generate *Wtap-*HKO mice. The genotype of *Wtap-*HKO mice is *Wtap*^flox/flox^ *Alb*-Cre^+/−^. All these mice were in C57BL/6J background. Male *Wtap*-HKO mice and *Wtap*^flox/flox^ control mice were given a single dose of DEN (50 mg/kg) intraperitoneally at 2 weeks old for DEN-induced HCC. The mice were randomly divided into several groups. *Wtap*-HKO and *Wtap*^flox/flox^ mice were fed an NC diet for 35 weeks before being killed for HCC investigation. For 11 weeks, the second and third groups of mice were fed an NC diet and HFD, respectively. Ki67 immunostaining has previously been demonstrated (10, 28).

### Primary hepatocyte culture and adenoviral infection

Primary hepatocytes were isolated from C57BL/6 WT, *Wtap*^flox/flox^, and *Wtap-*HKO mice *via* liver perfusion with type II collagenase (Worthington Biochem) and cultured in RPMI1640 medium supplemented with 3% fetal bovine serum at 37 °C in 5% CO_2_ incubator. Cells were infected with an equal amount of Ad-βGal, Ad-FLAG-PSMB4, or Ad-FLAG-PSMB6 adenoviruses overnight.

### Proteasome activity assay

Liver or hepatocyte samples were lysed in 50 mM Tris–HCl, pH 7.5, 250 mM sucrose, 5 mM MgCl_2_, 1 mM DTT, 2 mM ATP, 0.5 mM EDTA, and 0.025% digitonin for 5 min in ice. The lysate was cleared by centrifugation for 10 min at 12,000 rpm. Protein concentration was measured by bicinchoninic acid assay. Equal amounts of total protein were incubated in the assay buffer containing 50 mM Tris–HCl (pH 7.5), 5 mM MgCl_2_, 40 mM KCl, 1 mM DTT, 2 mM ATP, 50 μM Z-LLE-AMC (amino-4-methylcoumarin; for caspase-like activity) in the presence or the absence of the proteasome inhibitor MG132 (50 μM) for 30 min at 37 °C. The proteasome activity was recorded as a fluorescent signal of amino-4-methylcoumarin release.

### Real-time RT–qPCR

RT–qPCR was carried out as previously described (29). In summary, total RNA was isolated using the Roche TriPure Isolation Reagent (Manheim), and first-strand complementary DNA was generated using random primers and M-MLV reverse transcriptase (Promega). The Roche LightCycler 480 real-time PCR machine (Roche) was used for the RT–qPCR. Individual gene expression was normalized to 36B4 expression. Table S1 contains a list of real-time RT–qPCR primers.

### Translation efficiency assay

The translation efficiency experiment was carried out as previously described (18). In brief, 200 mg liver samples from *Wtap*^flox/flox^ and *Wtap-*HKO mice were isolated, immediately frozen, and pestled into powder in liquid nitrogen. About 600 μl polysome lysis buffer (20 mM Tris–HCl [pH 7.4], 150 mM NaCl¸ 5 mM MgCl2, 1% Triton-100, 1× Protease inhibitor cocktail, 1 mM DTT, 100 μg/ml cycloheximide, 1 U/μl SUPERase In RNase Inhibitor) was added. The samples were rotated and triturated before being centrifuged at 13,000 rpm for 10 min at 4 °C. The supernatant was analyzed for absorbance at 260 nm using NanoDrop 2000 before being transferred to a sucrose gradient (15%–55% sucrose gradient prepared by The Gradient Master) for ultracentrifugation at 38,000 rpm for 3 h at 4 °C. The Piston Gradient Fractionator was used to examine and collect ribosome fractions. Total RNA was isolated from each fraction, and RT–PCR was performed.

### ChIP assays

Liver perfusion with 1% paraformaldehyde was used to fix the livers. The nuclei were extracted from livers and sonicated (M220 Focused-ultrasonicator; Covaris) to break genomic DNA into 500- to 1000-bp fragments using a chromatin shearing kit (520127; truChIP Chromatin Shearing Kit; Covaris). Anti-RNA polymerase II antibodies (Rpb1 CTD, catalog no.: 2629; Cell Signaling Technology) were used to immunoprecipitate the samples. DNA was extracted, and qPCR analysis was performed. Table S1 contains a list of qPCR primers.

### Immunoprecipitation and immunoblotting

l-radioimmunoprecipitation assay lysis buffer (50 mM Tris, pH 7.5, 1% Nonidet P-40, 150 mM NaCl, 2 mM EGTA, 1 mM Na_3_VO_4_, 100 mM NaF, 10 mM Na_4_P_2_O_7_, and 1 mM PMSF) was used to homogenize cells or tissues. SDS-PAGE was used to separate the protein, which was then immunoblotted with the specified antibodies and detected with the ECL. The following antibodies were displayed: FLAG (catalog no.: F1804, 1:5000 dilution; Sigma); WTAP (catalog no.: 10200-1-AP, 1:2000 dilution; Proteintech); PSMB4 (catalog no.: 11029-1-AP, 1:5000 dilution; Proteintech); PSMB6 (catalog no.: 11684-2-AP, 1:5000 dilution; Proteintech); p-STAT3 (catalog no.: 9145, 1:3000 dilution; Cell Signaling Technology); STAT3 (catalog no.: 10253-2-AP, 1:3000 dilution; Proteintech); p-IκBα (Ser32) (catalog no.: 2859, 1:3000 dilution; Cell Signaling Technology); IκBα (catalog no.: 10268-1-AP, 1:2000 dilution; Proteintech); Tubulin (catalog no.: sc-5286, 1:5000 dilution; Santa Cruz); CD36 (catalog no.: 18836-1-AP, 1:2500 dilution; Proteintech); CCL2 (catalog no.: 66272-1-Ig, 1:2000 dilution; Proteintech); p-p65 (catalog no.: 3033, 1:2500 dilution; Cell Signaling Technology); p65 (catalog no.: 8242, 1:2500 dilution; Cell Signaling Technology); p-ERK1/2 (catalog no.: 4377, 1:5000 dilution; Cell Signaling Technology); ERK1/2 (catalog no.: 4695, 1:5000 dilution; Cell Signaling Technology); GRB2 (catalog no.: sc-8034, 1:1000 dilution; Santa Cruz); p-Met (catalog no.: 3077, 1:4000 dilution; Cell Signaling Technology); Met (catalog no.: 3127, 1:4000 dilution; Cell Signaling Technology); p-EGFR (catalog no.: 3777, 1:4000 dilution; Cell Signaling Technology); EGFR (catalog no.: 4267, 1:5000 dilution; Cell Signaling Technology); p-MEK1/2 (catalog no.: 9154, 1:4000 dilution; Cell Signaling Technology); MEK1/2 (catalog no.: 8727, 1:4000 dilution; Cell Signaling Technology); and IGFBP1 (catalog no.: A11672, 1:3000 dilution; ABclonal).

### Statistical analysis

The data were reported as means ± SD. Student's *t* tests were used to compare the differences between two groups. The differences between three groups were examined using one-factor ANOVA, and the least significance difference *t* test. *P* <0.05 was considered statistically significant in all analyses. Kolmogorov–Smirnov test for normality with *p* >0.1 suggested the samples followed a normal distribution. Statistical analyses and figures were made using GraphPad Prism version 6.02 (GraphPad Software, Inc). ANOVA and least significance difference *t* test analyses were performed using SPSS 21.0 (SPSS, Inc). ∗*p* <0.05 and ∗∗*p* <0.01.

## Data availability

ATAC-Seq, RNA-Seq, ChIP-Seq, and methylated RNA immunoprecipitation sequencing data that support the findings of this study have been deposited in Gene Expression Omnibus under accession codes GSE168945, GSE168850, GSE198023, and GSE192884, respectively. All other data are available from the authors upon request.

## Supporting information

This article contains supporting information.

## Conflict of interest

The authors declare that they have no conflicts of interest with the contents of this article.
