# Supplementary material for: Hepatocyte-specific Wtap deficiency promotes hepatocellular carcinoma by activating GRB2–ERK depending on downregulation of proteasome-related genes
Source: J Biol Chem. 2023 Sep 28;299(11):105301. doi: 10.1016/j.jbc.2023.105301 (PMC10630636; doi:10.1016/j.jbc.2023.105301)
Supplement: Supporting Figures S1–S3 and Tables S1 [file mmc1.docx]

**Supplementary Figures and Figure legends**


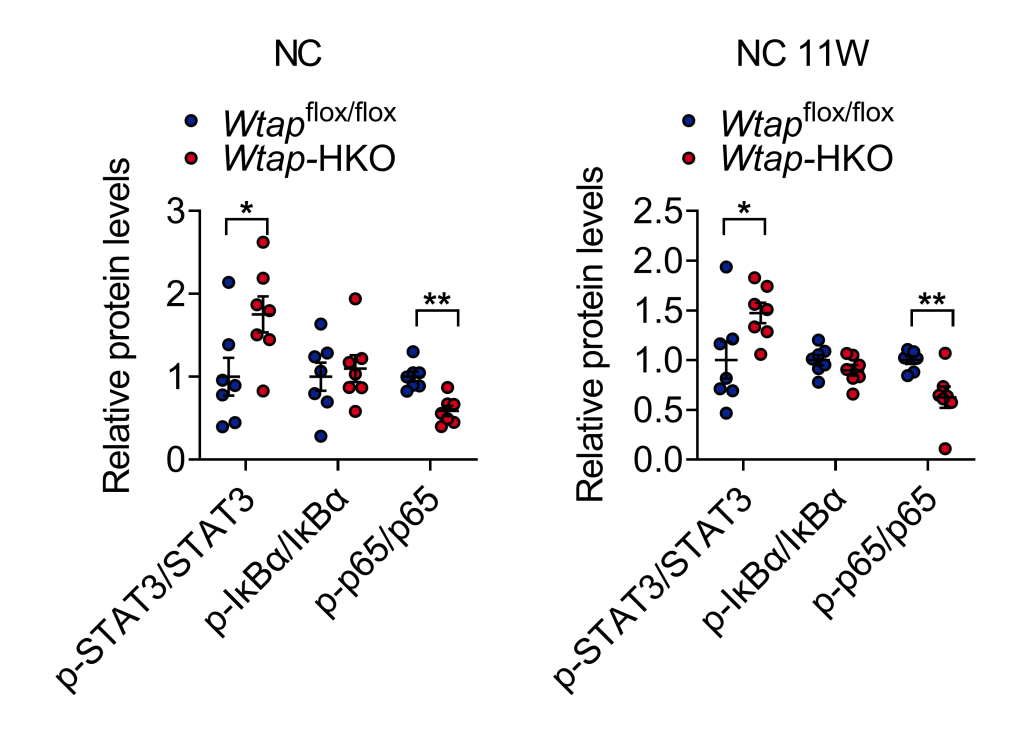


**Fig. S1 *Wtap*** **deletion in hepatocytes raises p-STAT3 but not p-IκBα or p-p65.**

Immunoblotting analysis of p-STAT3, STAT3, p-IκBα, IκBα, p-p65 and p65 in the livers of NC-fed *Wtap*-HKO and *Wtap*^flox/flox^ mice treated with or without DEN (*n*=7 for each group; NC: p-STAT3/STAT3, *p*=0.0341; p-IκBα/IκBα, *p*=0.6858; p-p65/p65, *p*=0.0003; NC DEN: p-STAT3/STAT3, *p*=0.0448; p-IκBα/IκBα, *p*=0.1931; p-p65/p65, *p*=0.0063). The representative images were shown in Figure 2L. Differences between two groups were analyzed by Student's t tests. *, *p*< 0.05. **, *p*< 0.01. Data represent the mean ± SD.

**Fig. S2 *Wtap* deletion in hepatocytes does not activate EGFR or Met.**

(A, D) Immunoblotting and Image J analysis of p-EGFR, EGFR, p-Met, Met, Gab1, SH-PTP2, p-B-Raf, B-Raf, and Tubulin in the livers of *Wtap*-HKO and *Wtap*^flox/flox^ mice fed NC for 8 weeks (*n*=7 for each group; *n*=4 for representative images; p-EGFR/EGFR: *p*=0.00245; p-Met/Met: *p*=0.0559; Gab1/Tubulin: *p*=0.0103; SH-PTP2/Tubulin: *p*=0.637; p-B-Raf/B-Raf: *p*=0.0302). (B-C, E-F) Immunoblotting and Image J analysis of p-EGFR, EGFR, p-MET, MET, Gab1, SH-PTP2, p-B-Raf, B-Raf, and Tubulin in the livers of *Wtap*-HKO and *Wtap*^flox/flox^ mice fed NC or HFD for 11 weeks following DEN treatment (*n*=7 for each group; *n*=4 for representative images; DEN NC 11W: p-EGFR/EGFR, *p*=0.8939; p-Met/Met, *p*=0.06989; Gab1/Tubulin, *p*=0.0109; SH-PTP2/Tubulin, *p*=0.01445; p-B-Raf/B-Raf, *p*=0.1587; DEN HFD 11W: p-EGFR/EGFR, *p*=0.3629; p-Met/Met, *p*=0.5007; Gab1/Tubulin, *p*=0.09588; SH-PTP2/Tubulin, *p*=0.7118; p-B-Raf/B-Raf, *p*=0.09068). Differences between two groups were analyzed by Student's t tests. *, *p*< 0.05. Data represent the mean ± SD.

**Fig. S3** **RT-qPCR analysis of downregulated proteasome-related genes in the livers of 8-week-old *Wtap*^flox/flox^ and *Wtap-*HKO mice** (n=8-10 for each group; *Psmb4*: *p*=0.0002; *Psma3*: *p*=0.00026; *Psma5*: *p*=0.000389; *Psma6*: *p*<0.0001; *Psmb2*: *p*<0.0001; *Psmb3*: *p*=0.005; *Psmb6*: *p*<0.0001**).** Differences between two groups were analyzed by Student's t tests. *, *p*< 0.05. **, *p*< 0.01. Data represent the mean ± SD.

| **Table S1 Primers for qPCR** | |  |
| --- | --- | --- |
| Genes | Forward | Reverse |
| *Psmb4* | 5'-CTACAGAGATGCCCGTTCGT-3' | 5'-TTGAGCCAGCTACAGTCACG-3' |
| *Psmb6* | 5'-CCATACCGACGCAGAAGCTA-3' | 5'-ACCGAGCTCAGTGCAATAGT-3' |
| *Psma3* | 5'-CCATGAAGGCTGTGGAAAACA-3' | 5'-CCTGCAACTGCCATTCCAAC-3' |
| *Psma5* | 5'-CTGTTAACCCAGGTCCGGTG-3' | 5'-GGCCACACGATTGAAAGCAG-3' |
| *Psma6* | 5'-GTGCTACGGGGTGCAGAC-3' | 5'-GTCCTTTCCTCTGACAGCAACA-3' |
| *Psmb2* | 5'-TCGTGTACCCGGGAAGTGTC-3' | 5'-AACATCTTGTCATGATCGTCCTTCA-3' |
| *Psmb3* | 5'-TTCTCAGGCCATGCTGAACG-3' | 5'-TGGGACCAGAGCACAGGTTA-3' |
| *Erk1* | 5'-CAACCCAAACAAGCGCATCA-3' | 5'-TGTCGAAGGTGAATGGCTCC-3' |
| *Erk2* | 5'-ATGACCCAAGTGATGAGCCC-3' | 5'-GAGCCCTTGTCCTGACCAAT-3' |
| *Grb2* | 5'-GGTTGCTCTGTTGCTTCTGC-3' | 5'-CACACAATGCCACCCGTGA-3' |
| *Gapdh* | 5'-TGTGTCCGTCGTGGATCTGA-3' | 5'-TTGCTGTTGAAGTCGCAGGAG-3' |
| *36B4* | 5'-AAGCGCGTCCTGGCATTGTCT-3' | 5'-CCGCAGGGGCAGCAGTGGT-3' |
| *Psmb4*-ChIP | 5'-CTCAGAGCCGGCCAATAGAG-3' | 5'-GCCATCTCTCCCCTTGGTTT-3' |
| *Psmb6*-ChIP | 5'-TGTGTAACGGTGGAAACCCT-3' | 5'-TGGAATGGTCAGGTCGTGTT-3' |
| *Actb*-ChIP | 5'-AATAGCCTCCGCCCTTGTG-3' | 5'-CGTGACATCCACACCCAGA-3' |
