# Supplementary material for: Hepatocyte-specific Wtap deficiency promotes hepatocellular carcinoma by activating GRB2–ERK depending on downregulation of proteasome-related genes
Source: J Biol Chem. 2023 Sep 28;299(11):105301. doi: 10.1016/j.jbc.2023.105301 (PMC10630636; doi:10.1016/j.jbc.2023.105301)
Supplement: Supporting information [file mmc2.pdf]

Data presentation check list:

This checklist is intended to help authors submit manuscripts that present data and figures in a transparent manner that is compatible with JBC guidelines and best practices in the field.

1. Have you reviewed the JBC instructions of authors and guidelines on data presentation?

   (●) Yes
   ( ) No *(Please review the following resources)*
   Author Information: https://www.jbc.org/content/authorinfo#idp2844848
   Best Practices for Data Presentation: http://jbcresources.asbmb.org/

2. Do any figures show images of gels or blots?

   (●) Yes
   ( ) No *(Please skip to question 3)*

   Click "Blot Images and Quantification" button on JBC guidelines web page for information.

   a) Are all the original data available on demand for review (or publicly available)?
      (●) Yes
      ( ) No
   b) Do the methods clearly describe how gel/blot images were developed and captured?
      (●) Yes
      ( ) No
   c) Are the full gels shown?
      (●) Yes
      ( ) No
      Are MW markers above and below the band(s) of interest indicated?
         (●) Yes
         ( ) No
         If no, please provide justification

      Are lanes cropped out of images presented?
         ( ) Yes
         (●) No
         Was the splicing position clearly indicated in the figures?
            ( ) Yes
            ( ) No
         If no, please provide justification

   d) Do the methods define how the specificity of each antibody used was validated?
      (●) Yes
      ( ) No

e) Are immunoblots for post-translational modifications (PTMs) (phosphorylation, methylation, etc) accompanied by parallel blots for total protein levels?

- (●) Yes
- ( ) No
- ( ) Not applicable

If no, please provide justification

f) Are the gels/blots quantitatively analyzed?

- (●) Yes
- ( ) No *(Please skip to question 3)*

Do the methods indicate how signals were quantified?

- (●) Yes
- ( ) No

Are signals normalized to the total protein loaded to each lane?

- (●) Yes
- ( ) No

If no, please provide justification

Are PTM signals normalized to the total level of the protein of interest?

- (●) Yes
- ( ) No

If no, please provide justification

3. Do any figures contain images of cells/tissues or similar images?

- (●) Yes
- ( ) No *(Please skip to question 4)*

Click "Image Data" button on JBC guidelines web page for information.

a) Is a scale bar shown in the image and defined in the figure legend?

- (●) Yes
- ( ) No

b) Do the methods define how the specificity of each antibody used was validated?

- (●) Yes
- ( ) No
- ( ) Not applicable

c) Are images depicting a single color shown in gray scale (…for the color blind)?

- (●) Yes
- ( ) No
- ( ) Not applicable

d) Are colors used in multi-color images selected to be accessible to the color blind.

- ◉ Yes
- ○ No
- ○ Not applicable

https://www.ascb.org/science-news/how-to-make-scientific-figures-accessible-to-readers-with-color-blindness/
https://journals.plos.org/plosbiology/article/file?type=printable&id=10.1371/journal.pbio.3001161

4. Do any of the figures contain bar graphs?

- ◉ Yes
- ○ No *(Please skip to question 5)*

Click "Quantitative Data and Statistics" button on JBC guidelines web page for information.

a) Are the results plotted as the mean ± S.D.

- ◉ Yes
- ○ No

If no, please provide justification

b) Are individual data points super-imposed on the bar graph?

- ◉ Yes
- ○ No

If yes, does the figure legend explicitly define individual data points as technical replicates (i.e., assay duplicates/triplicates, etc) or independent biological replicates?

- ◉ Yes
- ○ No

If no, please provide justification

5. Do the conclusions depend on comparisons between multiple quantitative measurements?

- ◉ Yes
- ○ No *(Please skip to question 6)*

Click "Quantitative Data and Statistics" button on JBC guidelines web page for information.

a) Is the statistical significance of differences between results/groups evaluated?

- ◉ Yes
- ○ No *(Please skip to question 6)*

b) Are data tested for a normal distribution?

- ◉ Yes
- ○ No *(Please skip to question c)*

Do the methods describe how?

- ◉ Yes
- ○ No

c) Are any data sets tested for statistical outliers?

- ○ Yes
- ◉ No *(Please skip to question d)*

Do the methods describe how?

- ○ Yes
- ◉ No

d) Is the specific statistical tests used in each panel defined in each figure legend?

- ◉ Yes
- ○ No

e) Is the specific type of t test (paired/unpaired; 1/2-tailed, one sample) defined for each use?

- ◉ Yes
- ○ No
- ○ Not applicable

f) Are multiple data sets compared in any figure panel?

- ◉ Yes
- ○ *No (Please skip to question 6)*

Is a 1-way or 2-way ANOVA used as needed for the number of variables?

- ◉ Yes
- ○ No

Does each figure legend report the full results of the ANOVA?

- ◉ Yes
- ○ No

Does each figure legend identify the post hoc testing method used?

- ○ Yes
- ○ No

Are exact p values indicated in the figure or figure legend?

- ◉ Yes
- ○ No

6. Were any of the experiments performed in cell lines?

- ○ Yes
- ◉ No *(Please skip the following questions)*

a) Do you describe the source/origin of cell line(s)?

- ○ Yes
- ○ No

b) Do you indicate whether and how the authenticity of the cell line(s) was validated (using STR profiling or other methodology)?

- ○ Yes
- ○ No

c) Do you indicate whether cell lines were tested for mycoplasma, and if cell lines were free from mycoplasma contamination for all experiments?

- ◯ Yes
- ◯ No

d) Do you suppress the expression of specific endogenous target genes in the cell line(s)?

- ◯ Yes
- ◯ No *(Please skip the following questions)*

Click "Gene Expression and Genetic Manipulation" button on JBC guidelines web page for information.

i. Do you use sh/siRNAs to manipulate gene expression?

- ◯ Yes
- ◯ No *(Please skip to question ii)*

Do you provide the sequences of the sh/siRNAs used?

- ◯ Yes
- ◯ No

Please indicate the controls used by checking the appropriate boxes. Do you:

- ☐ Use two si/shRNAs targeting different portions of the target gene?
- ☐ Use a control siRNAs with sequences altered from the target?
- ☐ Verify depletion of the targeted protein?
- ☐ Functionally rescue effects of knockdown by re-expressing the target gene?

ii. Do you use CRISPR to target a specific endogenous gene?

- ◯ Yes
- ◯ No *(Please skip the following questions)*

Do you provide the sequences of all guide RNAs?

- ◯ Yes
- ◯ No

Please indicate the controls used by checking the appropriate boxes.

- ☐ Use two or more clones of the manipulated cell lines?
- ☐ Use two or more guide RNAs targeting different portions of the target gene?
- ☐ Use control isogenic cells that retain a wild-type allele?
- ☐ Verify depletion (or mutation) of the targeted protein?
- ☐ Functionally rescue effects of the manipulation by re-expressing the target gene?
